# Fluvoxamine may reverse the decrease in filtering capacity that occurs in acute kidney injury by increasing IL-10 and ACE expressions and preserving AQ-2, AQ-4, CL-5, and ZO-1 expressions

**DOI:** 10.1007/s00210-025-03844-2

**Published:** 2025-01-29

**Authors:** Nasif Fatih Karakuyu, Tugce Camlica, Halil Asci, Adem Milletsever, Arzu Ulusoy, Fatih Mehmet Gonuler, Atila Altuntas

**Affiliations:** 1 Faculty of Pharmacy, Department of Pharmacology, Suleyman Demirel University, Isparta, Turkey; 2 Health Research and Application Center, Suleyman Demirel University Research and Application Hospital, Isparta, Turkey; 3 Faculty of Medicine, Department of Pharmacology, Suleyman Demirel University, Isparta, Turkey; 4 Faculty of Veterinary Medicine, Department of Pathology, Burdur Mehmet Akif Ersoy University, Burdur, Turkey; 5 Institute of Science, Department of Bioengineering, Suleyman Demirel University, Isparta, Turkey; 6 Faculty of Medicine, Suleyman Demirel University, Isparta, Turkey; 7 Division of Nephrology, Faculty of Medicine, Department of Internal Medicine, Suleyman Demirel University, Isparta, Turkey

**Keywords:** Acute kidney injury, Inflammation, Oxidative stress, Rats

## Abstract

The kidneys have a regulatory role in many diseases with their diuresis function and capacity to maintain electrolyte balance. In case of extensive damage, the kidney’s filtration capacity is impaired and cannot fulfill its functions. Fluvoxamine (FLV), an antidepressant agent, has antioxidant and anti-inflammatory effects. This study aimed to investigate the protective effects of FLV against renal tissue damage in the lipopolysaccharide (LPS) induced systemic inflammation model. Thirty-two Wistar Albino rats were divided into four equal groups: control, LPS, LPS + FLV, and FLV alone. Six hours after LPS administration, rats were sacrificed and kidney tissues were removed. Histopathological examination of kidney tissues, immunohistochemical expression of the ACE-1, Cas-9, and interleukin-10 (IL-10), as well as spectrophotometric assessment of total oxidant status (TOS), total antioxidant status (TAS), oxidative stress index (OSI), urea, creatinine levels, and zonulin-1 (ZO-1), claudin-5 (CL-5), aquaporins 2 and 4 (AQ-2, AQ-4) were evaluated. TOS, OSI, urea, creatinine levels, and Cas-9 expressions were significantly increased, and TAS levels, ACE-1, IL-10, AQ-2, AQ-4, ZO-1, and CL-5 expressions significantly decreased in the LPS group compared with the control group. In addition, marked hyperemia, slight-to-moderate hemorrhages, and degeneration of the tubule epithelial cells were observed in kidney sections in the LPS group, in addition to the inflammatory cell infiltrations. FLV treatment significantly improved all these findings. FLV treatment ameliorates renal tissues against inflammatory scenes and protects renal function by enhancing IL-10 and ACE-1 expressions and preserving AQ-2, AQ-4, CL-5, and ZO-1 expressions.

**Graphical Abstract:**

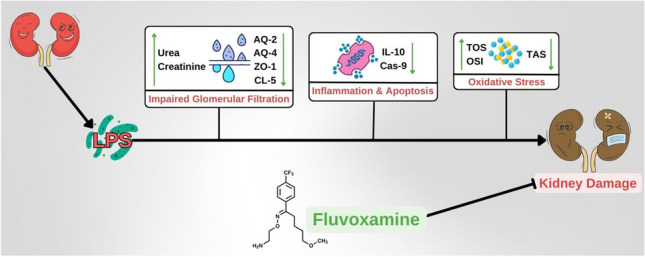

**Supplementary Information:**

The online version contains supplementary material available at 10.1007/s00210-025-03844-2.

## Introduction

In the case of central nervous system diseases, cytokines released from the intense inflammatory table caused by damage to the brain tissue can cross the blood–brain barrier through the blood and trigger damage in peripheral organs with plenty of blood supply (Galea 2021). Cytokines reaching the kidney tissue can stimulate damage mechanisms such as inflammation, oxidative stress, and apoptosis through their receptors, leading to clinical problems due to progressive kidney damage and increased urea and creatinine in the blood (Galea 2021; Ho and Shirakawa 2022).

This organ may be important in maintaining blood pressure in the human body through its diuresis function, and it has a regulatory role in many diseases through its capacity to maintain electrolyte balance. Possible damage to the tissue can affect all these functions and aggravate the patient’s condition. In the kidney, ACE-1 has an important role in stabilizing blood pressure as part of the renin-angiotensin system (Amatruda et al. 2022; Ho and Shirakawa 2022).

In diuresis, which is the most important task of the kidney, the mechanism of excretion is maintained by opening the intercellular spaces for the excretion of excess fluids in the body and by increasing the water canals in the protein structure called aquaporin (Bailey 2016; Natochin 2021; Ho and Shirakawa 2022). Biomarkers such as ZO-1 and CL-5, which are proven to be expressed in the kidney, show this increase in intercellular permeability, while AQ-2 and AQ-4 are important water channels. In case of extensive damage, damage or a decrease in the number of these structures will impair the filtration capacity of the kidney and cause it to be unable to perform its functions (Portincasa et al. 2021; Hassan et al. 2024).

FLV, a selective serotonin reuptake inhibitor, is an effective agent used in the treatment of depression. The antioxidant and anti-inflammatory effects of this agent, which can also be used in the treatment of depression that usually accompanies other organ pathologies, have been proven. These anti-inflammatory effects are achieved through several mechanisms, including nuclear factor kappa-light-chain-enhancer of activated B cells pathway modulation, NLR family pyrin domain containing three inflammasome inhibition, and reduction in pro-inflammatory cytokines such as interleukin-6 (IL-6), tumor necrosis factor-alpha, and interleukin-1β (IL-1β). The drug also enhances the activity of key antioxidant enzymes, including superoxide dismutase, catalase, and glutathione peroxidase. Research has shown that fluvoxamine treatment leads to increased levels of these endogenous antioxidant systems while simultaneously reducing markers of oxidative stress such as malondialdehyde and other lipid peroxidation products (Sukhatme et al. 2021; Ahsan et al. 2023).

This study aimed to investigate the protective effects of FLV against renal tissue damage in the LPS-induced systemic inflammation model and its effects on AQ-2, AQ-4, ZO-1, and CL-5 expressions.

## Materials and methods

### Ethical approval

The processes and procedures intended to be performed on rats within the research were reviewed and approved by the Local Ethics Committee of Süleyman Demirel University Animal Experiments (Ethics No: 11.07.2024/08–315). All methods were performed in accordance with the relevant guidelines and regulations. All experiments and procedures were performed per the Animal Research: Reporting in Vivo Experiments (ARRIVE) guidelines. In addition, this study was supported by the Scientific Research Projects Coordination Unit of Suleyman Demirel University with the project code TSG-2024–9268.

### Animals and experimental design

A total of 32 adult (13-week-old) female Wistar albino rats weighing between 300 and 350 g were provided by Suleyman Demirel University Experimental Animals and Medical Research Application and Research Center. The rats were divided into four equal groups, and each group was housed in separate standard Euro-type four cages. The study environment was carefully regulated to maintain a temperature range of 21–22 °C and a humidity level of 55–65%. In addition, all rats were kept under a consistent 12 h of light and 12 h of dark cycle as part of the experimental conditions. Throughout the study, the rats were fed ad libitum with a standard commercial feed and provided with water. The four experimental groups were formed as follows:

1. ***Control group (n = 8):*** 0.5–1 mL of saline was administered by oral gavage for 3 days, and 30 min after the last treatment, 0.5 mL of saline was administered i.p. in the right inguinal region.
2. ***LPS group (n = 8):*** 0.5–1 mL of saline was administered by oral gavage for 3 days, and 30 min after the last treatment, 5 mg/kg of LPS (L2630, Sigma-Aldrich, Germany) in 0.5 mL volume dissolved in saline was administered i.p. in the right inguinal region (Zhao et al. 2016; Cao et al. 2017).
3. ***LPS + FLV group (n = 8):*** 50 mg/kg/day FLV (Faverin, Abbott, Turkey) in 0.5–1 mL volume in saline was administered by oral gavage for 3 days (Yokoyama et al. 2007; Hajhashemi et al. 2008; Dursun et al. 2009; Rafiee et al. 2016); 30 min after the last treatment, 5 mg/kg of LPS in 0.5 ml volume was administered i.p. in the right inguinal region.
4. ***FLV group (n = 8):*** FLV at a dose of 50 mg/kg in 0.5–1 mL volume in SF was administered by oral gavage for 3 days; 30 min after the last treatment, 0.5 mL of SF was administered i.p. in the right inguinal region.

Rats were euthanized 6 h after the last drug administration under 90 mg/kg of ketamine (Keta-Control, Doğa İlaç, Turkey) and 8–10 mg/kg of xylazine (Xylazinbio 2%, Bioveta, Czech Republic) anesthesia. Following the abdominal incision, euthanasia was performed by surgical exsanguination with blood taken from the vena cava inferior. Kidney tissues were removed and portioned. Half of the kidney tissues were put into a 10% formaldehyde solution for histopathological and immunohistochemical analysis as ACE-1, Cas-9, and IL-10. The other halves were stored at − 80 °C for biochemical and relative mRNA expression analysis.

### Biochemical examination

Kidney tissues obtained for oxidative stress parameters were portioned, placed in Eppendorf tubes, and stored at − 80 °C until the analysis day. Then, tissues were diluted five-fold (w/v) with phosphate-buffered saline (10 mM sodium phosphate) at pH 7.4 and homogenized using a tissue homogenizer (IKA Ultra Turrax T25, Janke & Kunkel, Staufen, Germany). After homogenization, the samples were centrifuged at 2000 rpm/20 min/ + 4 °C (Nuve NF 1200R, Ankara, Turkey). Subsequently, they were transferred to the SDU Faculty of Medicine Biochemistry Laboratory for triple measurements. To assess oxidative stress, the levels of TAS and TOS were analyzed using the spectrophotometric method (Savran et al. 2020). TOS results were µmol H_2_O_2_/g protein, and TAS results were mmol Trolox Eq/g protein. The OSI was calculated by dividing the TOS levels by TAS levels, that is, TOS/TAS/10.

The collected rats’ blood was centrifuged at 3000 RPM for 15 min, and serum urea levels and creatinine levels were measured spectrophotometrically with a Beckman Coulter AU5800 autoanalyzer (Beckman Coulter, USA).

### Histopathological examination

Kidney tissue samples were collected during necropsy and fixed in 10% neutral formalin. The samples were then processed using an automatic tissue processor (Leica ASP300S, Wetzlar, Germany) and embedded in paraffin wax. Sections of 5-micron thickness were cut from the paraffin blocks with a rotary microtome (Leica RM2155, Leica Microsystems, Wetzlar, Germany). The sections were stained with hematoxylin–eosin (HE), mounted with coverslips, and examined under a light microscope (Olympus CX41, Tokyo, Japan). Histological lesions in the kidney tissues were semi-quantitatively scored for hyperemia, hemorrhage, inflammatory cell infiltration, and degenerative and necrotic changes (Supplementary Table S1).

### Immunohistochemical examination

For immunohistochemical analysis, kidney sections from each block were stained on poly-L-lysine coated slides for ACE-1, Cas-9, and IL-10 expression using the streptavidin–biotin technique with antibodies from Abcam (Cambridge, UK). Sections were incubated with primary antibodies for 60 min, followed by staining with a biotinylated secondary antibody and streptavidin–alkaline phosphatase conjugate. The EXPOSE Mouse and Rabbit Specific HRP/DAB Detection IHC kit (Abcam) was used for secondary antibody detection, with DAB as the chromogen. Negative controls utilized antigen dilution solution instead of the primary antibody. A specialized pathologist from another university, blinded to the study, conducted all examinations.

Each section was examined separately for immunohistochemical analysis. To evaluate the severity of the immunohistochemical reaction of cells with markers, semiquantitative analysis was performed using a grading score ranging from (0) to (3) (Supplementary Table S1).

Each section was examined in 10 different areas under 40X objective magnification for evaluation. Morphometric analyses and microphotography were performed using the Database Manual Cell Sens Life Science Imaging Software System (Olympus Co., Tokyo, Japan). The results were saved and statistically analyzed (Candan et al. 2024).

### Reverse transcription-polymerase chain reaction (RT-qPCR)

Following the manufacturer’s protocol, RNA was extracted from homogenized tissues using the GeneAll RiboEx™ RNA Isolation Kit (GeneAll Biotechnology, Korea). The concentration and purity of the isolated RNA were assessed using the BioSpec-nano nanodrop device (Shimadzu Ltd., Japan). For cDNA synthesis, 1 µg of RNA was utilized. The cDNA synthesis was performed with the cDNA Synthesis Kit (Atlas Biotechnology, Turkey) in a thermal cycler, adhering to the manufacturer’s instructions. Specific mRNA sequences were identified, and potential primer sequences were designed and validated using the NCBI website (Supplementary Table S2). Gene expression levels were quantified using a Biorad CFX96 real-time PCR system (USA) with 2X SYBR Green master mix (Nepenthe, Turkey). GAPDH served as the housekeeping gene for normalization. The reaction mixture was prepared following the manufacturer’s protocol, resulting in a final volume of 20 µL per reaction. The reactions were run in triplicate on a real-time qPCR machine according to the kit’s instructions. The RT-qPCR cycling conditions included an initial denaturation at 94 °C for 10 min (1 cycle), followed by 40 cycles of denaturation at 95 °C for 15 s, and annealing/extension at 55 °C for 30 s. Relative mRNA expression levels were determined using the 2-ΔΔCt method after normalization to the housekeeping gene (Dogan Unlu et al. 2024).

### Statistical analysis

Statistical analyses were evaluated with the Graphpad Prism 8.0 program pack (Graphpad Software. Inc., USA). In addition to descriptive statistical methods (mean, standard deviation), the one-way ANOVA Tukey test was used to evaluate the study data. The significance level was taken as *p* < 0.05.

## Results

### Biochemical results

**Fig. 1:**
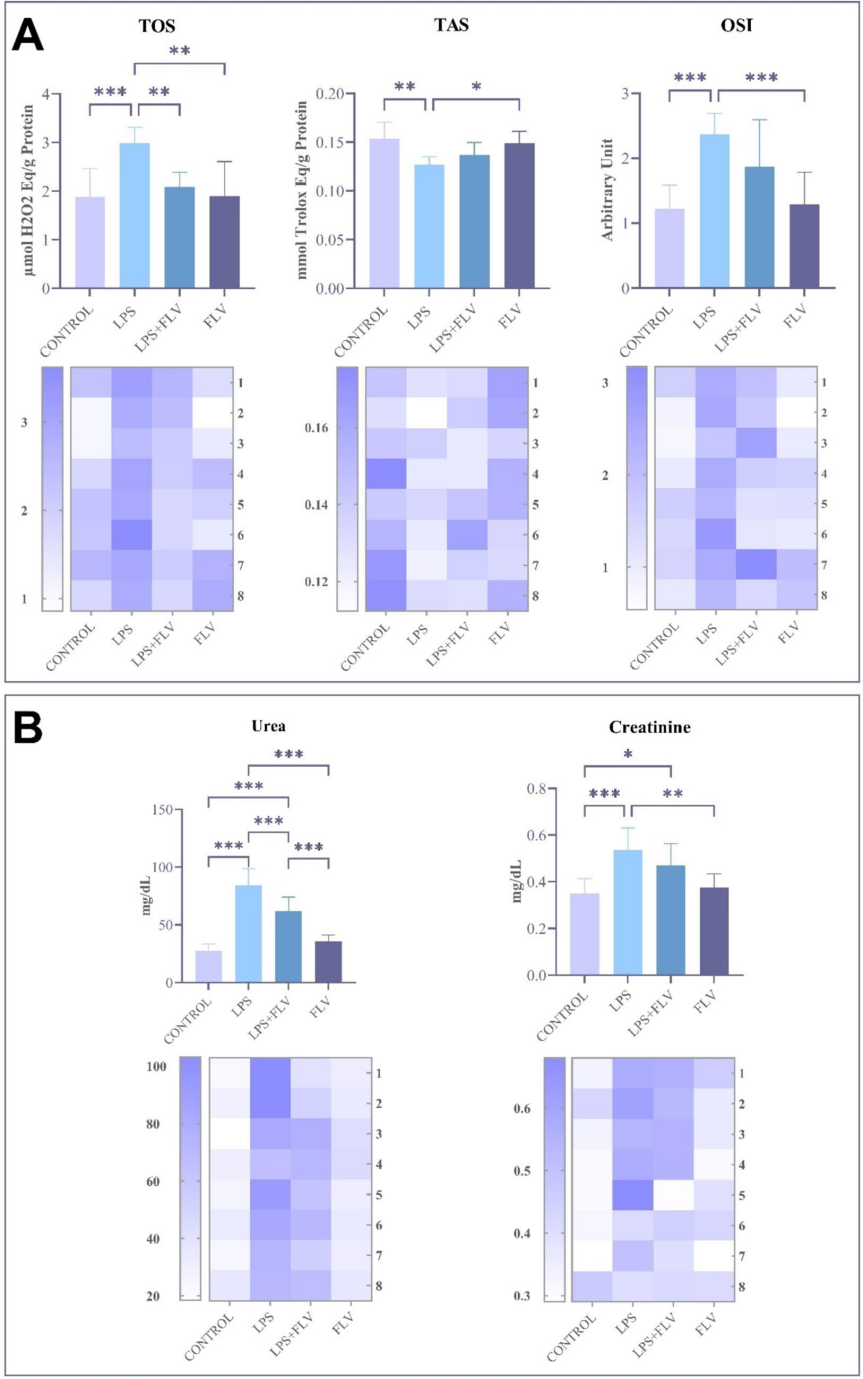
Biochemical parameters of this study. LPS: lipopolysaccharide, FLV: fluvoxamine, TOS: total oxidant status, TAS: total antioxidant status, OSI: oxidative stress index. Values are presented as means ± standard deviation. *p ≤ 0.05, **p ≤ 0.01, ***p ≤ 0.001

TOS, TAS, and OSI values of the rats evaluated within the research according to the groups are presented in Fig. 1A. A statistically significant difference was found between the LPS group and the control (*p* ≤ 0.001), FLV (*p* ≤ 0.01), and LPS + FLV (*p* ≤ 0.01) groups when the TOS values of the rats were compared. According to these differences, it was found that the TOS values of the rats in the LPS group increased more than the rats in the control, FLV, and LPS + FLV groups. When the TAS values of the rats were compared, a statistically significant difference was found between the control group and LPS (*p* ≤ 0.01) groups and between LPS and FLV (*p* ≤ 0.05) groups. According to these differences, it was determined that the TAS values of the rats in the LPS group decreased more than the rats in the control group, and the TAS values of the rats in the LPS group decreased more than the rats in the FLV group. When the OSI values of the rats were compared, a statistically significant difference was found between the LPS group and the control (*p* ≤ 0.001) and FLV (*p* ≤ 0.001) groups. According to these differences, it was found that the OSI values of the rats in the LPS group increased more than the rats in the control and FLV groups (Fig. 1).

The urea and creatinine values of the rats evaluated within the research according to the groups are presented in Fig. 1B. When urea values of rats were compared, a statistically significant difference was found between the control group and LPS (*p* ≤ 0.001) and LPS + FLV (*p* ≤ 0.001) groups; between the LPS group and FLV (*p* ≤ 0.001) and LPS + FLV (*p* ≤ 0.001) groups; between FLV group and LPS + FLV (*p* ≤ 0.001) group. When creatinine values of rats were compared, a statistically significant difference was found between the control group and LPS (*p* ≤ 0.001) and LPS + FLV (*p* ≤ 0.05) groups; between the LPS group and FLV (*p* ≤ 0.01) group. According to these differences, it was determined that the urea values of the rats in the LPS group increased more than the rats in the control, FLV, and LPS + FLV groups, and the urea values of the rats in the LPS + FLV group increased more than the rats in the control and FLV groups. It was determined that the creatinine values of the rats in the LPS group increased more than the rats in the control and FLV groups (Fig. 1).

### Histopathological results

**Fig. 2:**
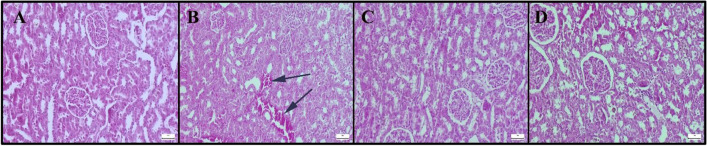
Histopathological appearance of kidney among the groups. **A** Normal tissue histoarchitecture in the control group, **B** slight hemorrhage (arrows) in the LPS group, **C** decreased pathological findings in the LPS + FLV group, **D** normal kidney histology in the FLV group, HE, scale bars = 50 µm

At the histopathological examination, marked hyperemia, slight-to-moderate hemorrhages, and degeneration of the tubule epithelial cells were observed in kidney sections in the LPS group, in addition to the inflammatory cell infiltrations. FLV treatment decreased pathological findings in the LPS + FLV group. Normal tissue histology was observed in the control and FLV groups (Fig. 2) (Supplementary Table S3).

### Immunohistochemical results

**Fig. 3:**
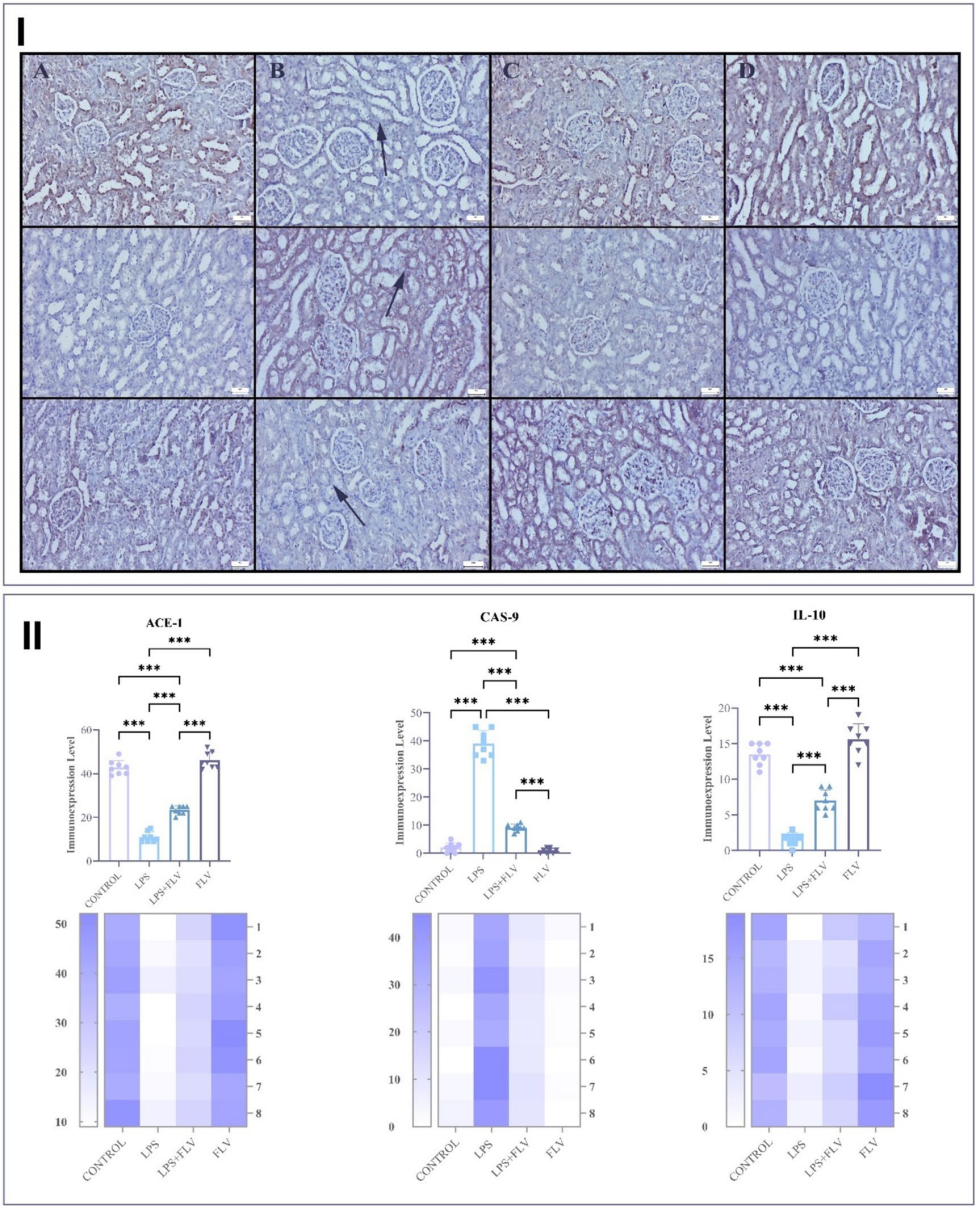
Immunohistochemical appearance of the kidney among the groups. **I –** ACE-1 (upper row), Cas-9 (medium row), and IL-10 (below row). **A** Marked ACE, moderate IL-10, and negative Cas-9 expression in the control group; **B** decreased ACE and IL-10 and increased Cas-9 expressions in tubular cells (arrows) in the LPS group; **C** increased ACE, decreased Cas-9, and increased IL-10 expressions in the LPS + FLV group; **D** marked ACE, slight IL-10, and negative Cas-9 expression in the FLV group. Streptavidin–biotin peroxidase method, scale bars = 50 µm. **II** – statistical analysis results. Values are presented as means ± standard deviation, *p ≤ 0.05, ***p ≤ 0.001

The immunohistochemical examination marked ACE-1 and IL-10 negative to slightly creased Cas-9, and expressions were observed in the tubular and glomerular cells in the control and FLV groups. LPS caused a decrease in ACE-1 and IL-10 while increasing Cas-9 expression in kidneys when compared to the control group (*p* ≤ 0.001). These expressions significantly reversed in the LPS + FLV group compared with the LPS group (*p* ≤ 0.001) (Fig. 3).

### Relative mRNA expression results

**Fig. 4:**
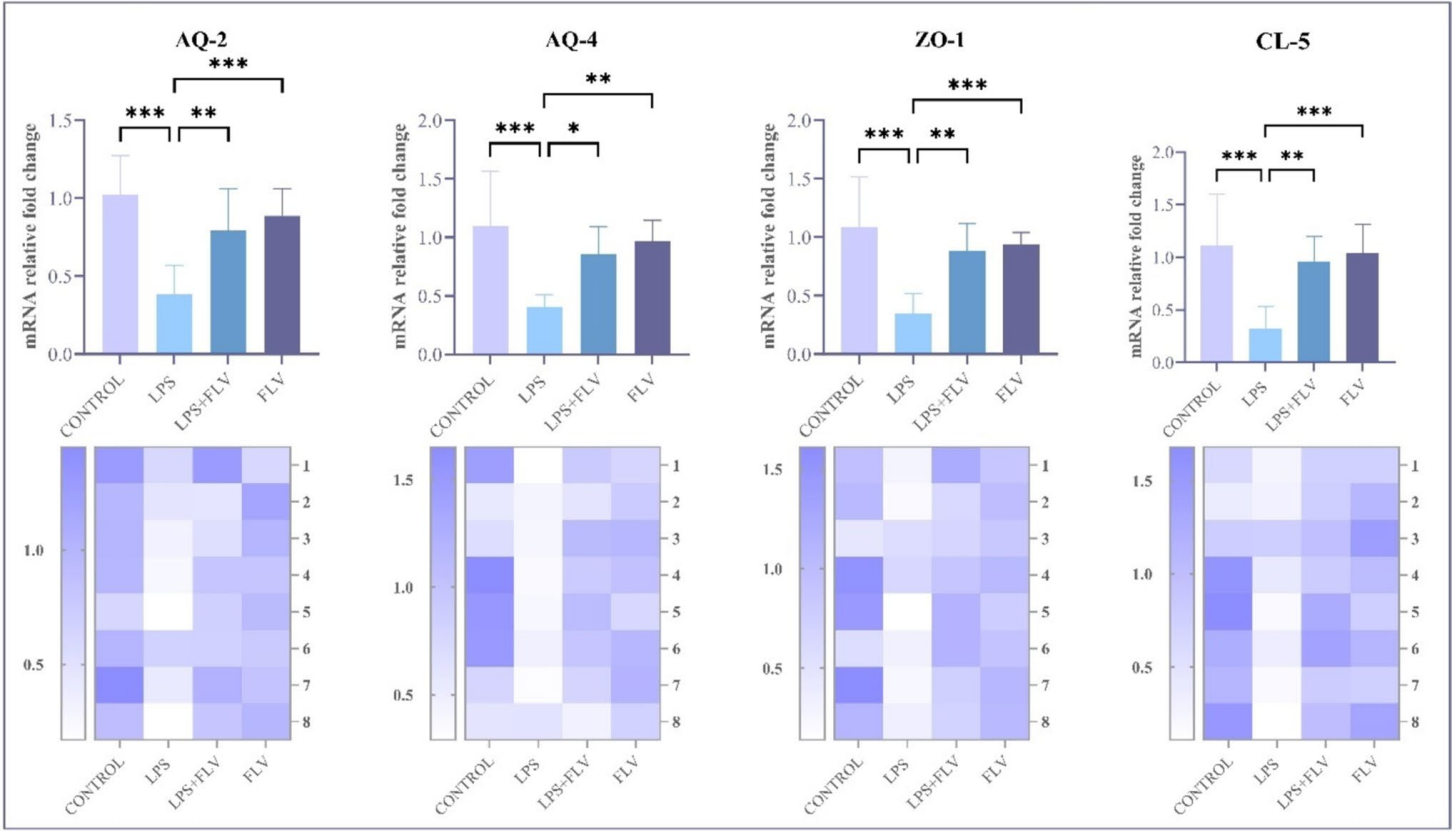
Genetical parameters of the kidney among the groups. AQ-2: aquaporin-2, AQ-4: aquaporin-4, CL-5: claudin-5, ZO-1: zonulin-1. LPS: lipopolysaccharide, FLV: fluvoxamine. Values are presented as means ± standard deviation. *p ≤ 0.05, **p ≤ 0.01, ***p ≤ 0.001

AQ-2, AQ-4, CL-5, and ZO-1 values of the rats evaluated within the research according to the groups were presented in Fig. 4. When the AQ-2 values of the rats were compared, a significant difference was found between the control group and LPS group (*p* ≤ 0.001), between the LPS group and FLV group (*p* ≤ 0.001), and between the LPS group and LPS + FLV group (*p* ≤ 0.01). When the AQ-4 values of the rats were compared, a significant difference was found between the control group and the LPS group (*p* ≤ 0.001), between the LPS group and FLV (*p* ≤ 0.01) and LPS + FLV (*p* ≤ 0.05) groups. When ZO-1 and CL-5 values of rats were compared, a statistically significant difference was found between the control group and LPS groups (*p* ≤ 0.001) and between the LPS groups and FLV (*p* ≤ 0.001) and LPS + FLV (*p* ≤ 0.01) groups (Fig. 4).

## Discussion

This study investigated the protective effect of FLV, a selective serotonin reuptake inhibitör agent, on LPS-induced renal injury and its effects on AQ-2 and AQ-4 water cannulae and ZO-1 and CL-5 cell membrane proteins required for diuresis.

In hospital conditions, patients hospitalized in intensive care units may develop additional diseases such as delirium, psychosis, or depression in addition to their main pathologies (Poulsen et al. 2021). For this reason, the need for multiple drug use arises with the accompaniment of polypharmacy and creates stress on the kidney tissue, which is the most important organ of excretion. The number of agents that can be directly applied for the protection of this organ, which can be acutely or chronically damaged, is very few. Therefore, it is expected that the drugs to be used in additional pathologies will also show protective activity in the kidney tissue (Dikeç and Dikeç 2021).

Different paradoxical responses can be seen in the organism during short-term and long-term drug use. While positive changes are observed in short-term use, undesirable effects can be seen in long-term use. Fluvoxamine may cause significant adverse changes in renal function by increasing serum creatinine levels with long-term use (Galal et al. 2016). However, there are also studies in which FLV, which can be used in depression accompanying major organ disorders, is applied in an acute injury model, and its protective effectiveness is proven (Hajhashemi et al. 2008; Barmoudeh et al. 2022). In the LPS-induced acute kidney injury model selected in this study, marked hyperemia, slight-to-moderate hemorrhages, inflammatory cell infiltrations, and degeneration of the tubule epithelial cells occurred in the tissue, and it was observed that the model was established. Anti-inflammatory cytokines such as IL-10, which are synthesized endogenously to suppress this inflammatory response, were observed to decrease in the LPS group in immunostaining. The reversal of this table, which resulted in a deterioration of the balance in favor of inflammation, with FLV treatment shows that the drug has anti-inflammatory activity. The increase detected in immunostaining only in the FLV-treated group compared to the control shows that the active substance has an isolated increase capacity on IL-10 and uses this feature in tissue protection. In order to increase the significance value, this increase should be followed up with higher or repeated doses.

It is also known that other types of damage can accompany inflammatory processes. It has been proven that the oxidant-antioxidant activity imbalance, also known as oxidative stress, causes cumulative aggravation of the damage (Barmoudeh et al. 2022; Ho and Shirakawa 2022; Ates et al. 2024). Increased TOS and OSI values and decreased TAS levels in the LPS group compared to the control showed that oxidative stress developed and was reversed by FLV treatment. The reversal of oxidative stress secondary to less inflammation caused by FLV by increasing IL-10 level in the tissue will also decrease; the antioxidant level to be used did not decrease excessively.

The inhibition by FLV of increased Cas-9 in the LPS group, which is an indicator of apoptosis secondary to mitochondrial organelle damage, which is parallel to both mechanisms and results in cellular death, indicates that the drug has an antiapoptotic effect and suppresses mitochondrial apoptosis.

The renin–angiotensin–aldosterone system is a very important mechanism, especially in the kidney tissue with an excretory function, and one of the enzymes catalyzing the reactions is ACE-1 (Amatruda et al. 2022; Ho and Shirakawa 2022). Decreased levels of this enzyme, necessary for the formation of angiotensin II, which causes the synthesis of aldosterone that retains water and sodium for endogenous fluid regulation, in the LPS group of the damage group, will cause disorders in water and sodium retention. It is important that these decreases in expression, which occur secondary to increased nephron damage, can be reversed by FLV because it reduces the damage. On the other hand, the increase in ACE-1 expression in the drug-alone group compared to the control group suggests that the drug may also have fluid retention properties in individuals with intact renal tissue, and this effect should be supported by more detailed molecular investigations in future studies.

Moreover, some structures have been proven to affect the filtration capacity of kidney tissue in recent years. These structures, such as ZO-1 and CL-5, which inhibit intercellular membrane permeability, show that the decrease in expression levels in case of damage causes an increase in permeability. On the other hand, through the presence of AQ-2 and AQ-4 canals in the membrane, which is known to allow the passage of water molecules, the passage of intercellular fluids can be provided, and diuresis can continue in the kidney tissue (Natochin 2021; Portincasa et al. 2021; Hassan et al. 2024). Diuresis problems occurring in the injury group are expected to be parallel with increased urea and creatinine levels in the blood, as in the clinic. In the present study, the increased urea and creatinine levels found in the damage group and the decreased AQ-2, AQ-4, ZO-1, and CL-5 expression determined by genetic analyses support this situation. The reversal of all these conditions with FLV treatment suggests that renal function may be preserved in acute kidney injury.

In conclusion, to prove this protective activity provided by FLV, the significance found in immune parameters should be supported by analyses such as western blot showing expression at the protein level, and the diuresis capacity of the rats should also be examined in a metabolic cage. Further investigations are also needed to identify other intracellular pathways that FLV might utilize to produce this effect. Moreover, future studies could focus on enhancing the bioavailability of fluvoxamine by developing it into different pharmaceutical forms.

## Supplementary Information

Below is the link to the electronic supplementary material.

Supplementary file1 (DOCX 19 KB)

## Data Availability

All source data for this work (or generated in this study) are available upon reasonable request.
