# Supplementary material for: Fluvoxamine may reverse the decrease in filtering capacity that occurs in acute kidney injury by increasing IL-10 and ACE expressions and preserving AQ-2, AQ-4, CL-5, and ZO-1 expressions
Source: Naunyn Schmiedebergs Arch Pharmacol. 2025 Jan 29;398(7):9021–9. doi: 10.1007/s00210-025-03844-2 (PMC12263809; doi:10.1007/s00210-025-03844-2)
Supplement: Supplementary file 1 — Supplementary file1 (DOCX 19 KB) [file 210_2025_3844_MOESM1_ESM.docx]

**Table S1.** Description of histopathologic scores and the severity of the immunohistochemical reaction

| 0 | Negative | (Normal) No damage |
| --- | --- | --- |
| 1 | Focal weak staining | (Mild) Mild hyperemia, slight hemorrhage, inflammation, and no degeneration |
| 2 | Diffuse weak staining | (Moderate) Severe hyperemia, slight hemorrhage, slight inflammation, and slight necrosis |
| 3 | Diffuse strong staining | (Severe) Severe hyperemia, severe hemorrhage, marked inflammation, and marked necrosis |

**Table S2.** Primary sequences, product size, and accession numbers of genes

| Genes | Primary Sequence | Product Size | Accession Number |
| --- | --- | --- | --- |
| GAPDH (HouseKeeping) | **F:** AGTGCCAGCCTCGTCTCATA | 248 bp | NM_017008.4 |
|  | **R:** GATGGTGATGGGTTTCCCGT |  |  |
| AQ-2 | **F:** CTGGGGGACTGTGCTTAGTG | 135 bp | NM_012909.3 |
|  | **R:** CCGGCTCTGTATCACCACAG |  |  |
| AQ-4 | **F:** TTGGACCAATCATAGGCGC | 212 bp | XM_039096587.1 |
|  | **R:** GTCAATGTCGATCACATGC |  |  |
| CL-5 | **F:** CCAACATCGTAGTCCGGGAG | 246 bp | NM_031701.2 |
|  | **R:** CCCGCCCTTAGACGTAGTTC |  |  |
| ZO-1 | **F:** ATCCCACAAGGAGCCATTCC | 185 bp | XM_039105296.1 |
|  | **R:** TCACAGTGTGGCAAGCGTAG |  |  |

*F: Forward, R: Reverse, GAPDH: Glyceraldehyde-3-phosphate dehydrogenase, AQ-2: Aquaporin-2, AQ-4: Aquaporin-4, CL-5: Claudin 5, ZO-1: Zonulin-1.*

**Table S3.** Histopathological examination scores of groups

|  | **Histopathological Examination Score**  **(Mean+SD)** |
| --- | --- |
| CONTROL | 0.00 ± 0.00 |
| LPS | 2.50 ± 0.53 |
| LPS+FLV | 0.62 ± 0.51 |
| FLV | 0.00 ± 0.00 |
